# Supplementary material for: Peculiarities of the Spatial and Electronic Structure of 2-Aryl-1,2,3-Triazol-5-Carboxylic Acids and Their Salts on the Basis of Spectral Studies and DFT Calculations
Source: Int J Mol Sci. 2023 Sep 12;24(18):14001. doi: 10.3390/ijms241814001 (PMC10531006; doi:10.3390/ijms241814001)
Supplement: Supplementary file 1 [file ijms-24-14001-s001.zip › ijms-2581391-supplementary.pdf]

SUPLEMENTARY MATERIAL

Peculiarities of the Spatial and Electronic Structure of 2-Aryl-1,2,3-Triazol-5-Carboxylic Acids and Their Salts on the Basis of Spectral Studies and DFT Calculations

Mauricio Alcolea Palafox [1,*], Nataliya P. Belskaya [2] and Irena P. Kostova [3]

[1] Departamento de Química Física, Facultad de Ciencias Químicas, Universidad Complutense, 28040 Madrid, Spain
[2] Department of Technology for Organic Synthesis, Ural Federal University, 19 Mira Str., 620012 Yekaterinburg, Russia; n.p.belskaya@urfu.ru
[3] Department of Chemistry, Faculty of Pharmacy, Medical University, 2 Dunav Str., 1000 Sofia, Bulgaria; irenakostova@yahoo.com
* Correspondence: alcolea@ucm.es

2.6 Vibrational Analysis of 1a and 2a

The C=O modes: The C=O out-of-plane bending is predicted (scaled) with medium-weak IR intensity in the monomer form at 705 cm^{-1}, and strongly coupled to γ(NCC)+γ(CN) modes. A large contribution of this mode was also predicted at 770 cm^{-1}. However, in the dimer form it is predicted with almost null IR and Raman intensity which difficult its determination in the spectra. The very low experimental IR band at 707.8 cm^{-1} and the weak Raman line at 705.9 cm^{-1} were assigned to this mode.

2.6.4 The aryl ring modes

The aromatic C-H stretching vibrations are generally observed in the 3200-3050 cm^{-1} range and they are predicted theoretically as almost pure modes (100% PED) with very weak and almost null IR intensity and weak Raman activity, in accordance to that observed experimentally. Mode **2** appears characterized as $\nu(C_3\text{-H})$ and scaled at similar wavenumbers in both molecules: at 3112 cm^{-1} in **1a** and 3106 cm^{-1} in **2a**. Mode **20b** is characterized as $\nu(C_5\text{-H})$ and scaled also at similar wavenumbers in accordance to the very weak effect of the COOH group on the benzene ring modes. Slight differences are only observed in modes **7b** and **20a**, where mode **7b**, in general characterized as $\nu(C_2\text{-H})$, is scaled at 3084 cm^{-1} in **1a**, and at 3066 cm^{-1} in **2a**, while mode **20a** is scaled at 3093 cm^{-1} in **1a** and at 3079 cm^{-1} in **2a**,

The aromatic C-C stretching vibrations, modes **8a** and **8b**, appears characterized as almost pure modes, with %PED higher than 90%, and they are observed in the spectra at similar wavenumbers. Mode **8a**, is predicted in both molecules with the highest Raman intensity. Thus, it is scaled in **1a** at 1625 cm^{-1} and good related to the very strong Raman line observed at 1615.2 cm^{-1}, while in **2a** is predicted at 1629 cm^{-1} and well related to the experimental Raman line at 1610.4 cm^{-1}. Mode **8b** is predicted with very weak IR intensity and weak-medium Raman activity in accordance to that observed in the spectra.

In modes **19a** and **19b** the motions appear characterized as CC+CH in accordance to benzene molecule [15] and their derivatives [11], and little coupled with other vibrations. Mode **19a** is predicted in 1a with strong IR and Raman intensity at 1520 cm^{-1}, in accordance to the very strong IR band at 1514.0 cm^{-1} and to the strong Raman line at 1511.1 cm^{-1}. In **2a** it is predicted with the strongest IR intensity in accordance to the very strong IR band observed experimentally at 1514.0 cm^{-1}, and it is also predicted with strong Raman intensity in good accordance to the very strong Raman line observed in the spectrum at 1515.9 cm^{-1}. Mode **19b** was predicted, by contrast, with weak intensity at 1438 cm^{-1} in agreement with the IR band detected experimentally at 1434.0 cm^{-1}.

The C-C stretching, mode **14**, appears coupled in 1a with ν_s(NNN) and predicted at 1345 cm^{-1} with weak-medium IR and Raman intensity in accordance to that observed in the experimental IR spectrum at 1333.7 cm^{-1}. In molecule **2a** it is coupled with $\delta(CH_3)$ and appears scaled at 1331 cm^{-1} and well related to the IR band observed at 1339.5 cm^{-1}. Because this mode **14** appears coupled with normal modes that are characterized at higher wavenumbers, its wavenumber is blue-shifted to a higher value than that reported in benzene molecule [15], calculated at 1341.7 cm^{-1} and scaled at 1309 cm^{-1} with the PSE procedure used in the present work.

Raman intensity calculations

Harmonic wavenumber computations were carried out at the same level of the respective optimization process and by the analytic evaluation of the second derivative of the energy with respect to nuclear displacement. The calculated Raman scattering activities (S_i) were converted into relative Raman intensities (I_i) using the following relationship derived from the basic theory of Raman scattering.

$$I_i = \frac{f\ (\nu_o - \nu_i)^4\ S_i}{\nu_i\left[1 - e^{-\left(\frac{hc\nu_i}{kT}\right)}\right]}$$

Where the symbols are defined as: ν_o: the frequency (cm^{-1}) of exciting radiation, ν_i: the vibrational frequency of the ith normal mode, h: Planck constant, c: speed of light, k: Boltzmann constant and T: absolute temperature, f: a suitably chosen scaling factor common to all the peak intensities.

Interaction energy calculations

The interaction energies were calculated in the dimer form of the molecules under study. These calculations were carried out only at the M06-2X/6-31G(d,p) level, and the values obtained were corrected for basis set superposition error, according to the procedure [40] which is as follow for the present study:

The total counterpoise (CP) corrected interaction energy of the dimer AA' is defined as $\Delta E^{CP}_{AA'}$ where AA' corresponds to both molecules of dimer, and it can be determined according to the following relation:

$$\Delta E^{CP}_{AA'} = E^{int}(AA') + E^{def}(AA') \qquad \{1\}$$

$$\text{where, } E^{int}(AA') = E^{AA'}_{AA'}(AA') - E^{AA'}_{A}(AA') - E^{AA'}_{A'}(AA') \qquad \{2\}$$

In this equation, $E^{AA'}_{AA'}(AA')$ corresponds to the calculated electronic energy at the optimized geometry of the dimer (AA'), and $E^{AA'}_{A}(AA')$ and $E^{AA'}_{A'}(AA')$ represents the electronic energy of each molecule, A and A' of the dimer (AA').

The deformation energy $E^{def}(AA')$ is described as: $E^{def}(AA') = E^{def}_{A}(AA') + E^{def}_{A'}(AA')$ {3}

Where $E_A^{def}(AA')$ and $E_{A'}^{def}(AA')$ of each monomer in the dimer can be determined by the following equations:

$$E_A^{def}(AA') = E_A^A(AA') - E_A^A(A)$$

and $$E_{A'}^{def}(AA') = E_{A'}^{A'}(AA') - E_{A'}^{A'}(A') = E_{A'}^{A'}(AA') - E_A^A(A) \qquad \{4\}$$

In these equations the parentheses indicate whether the computations in the dimer form were done at the optimized molecular geometry of each monomer form (A and A′) or that of the dimer (AA′). Because the optimized form of each monomer is the same, $E_A^A(A) = E_{A'}^{A'}(A')$.

The superscripts denote whether the calculations were done with the basis set of monomer or dimer, and the subscripts indicate the molecular system studied.

[40] Boys, S.F.; Bernardi, F. Calculation of small molecular interactions by differences of separate total energies. Some procedures with reduced errors. *Molec. Phys.*, **1970**, *19*(4), 553.

1a

Conf. 2.

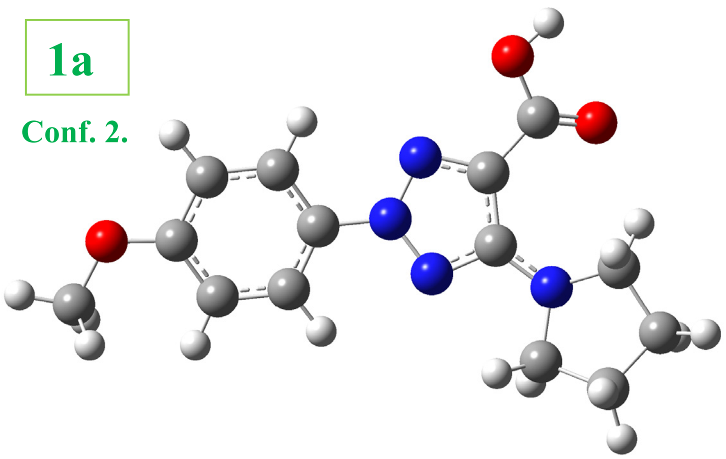

E(MP2+ZPE) = -984.622986 AU (G = -984.672646 AU)

Conf. 3.

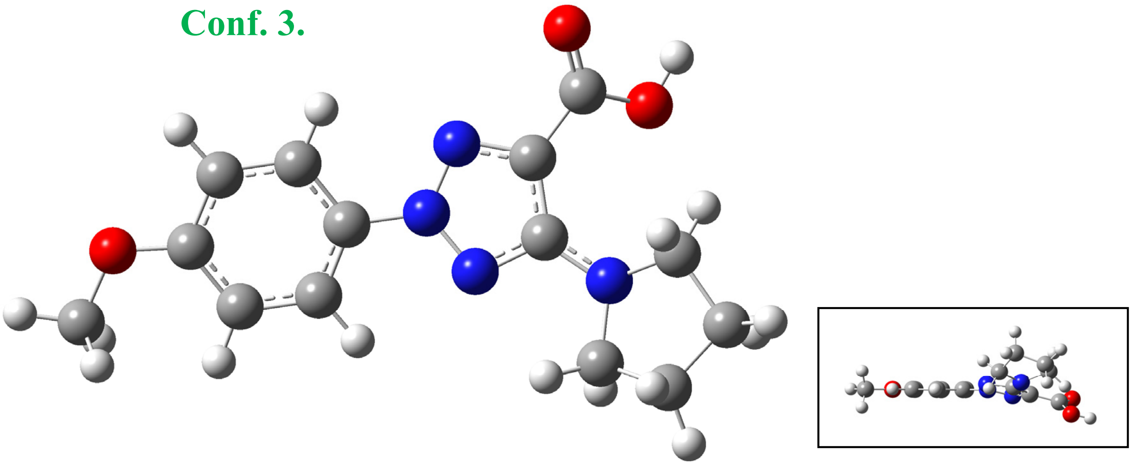

E(MP2+ZPE) = -984.619026 AU (G = -984.669435 AU)

Conf. 4.

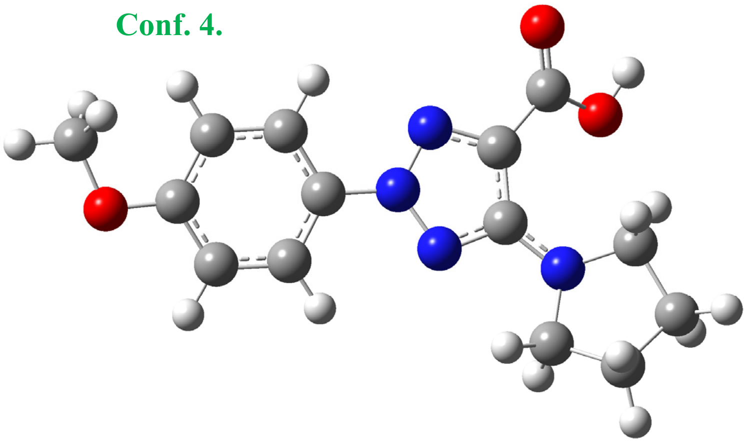

E(MP2+ZPE) = -984.619127 AU (G = -984.669555 AU)

Figure S1-SUP. Others optimized conformers of **1a** molecule at the MP2/6-31G(d,p) level.

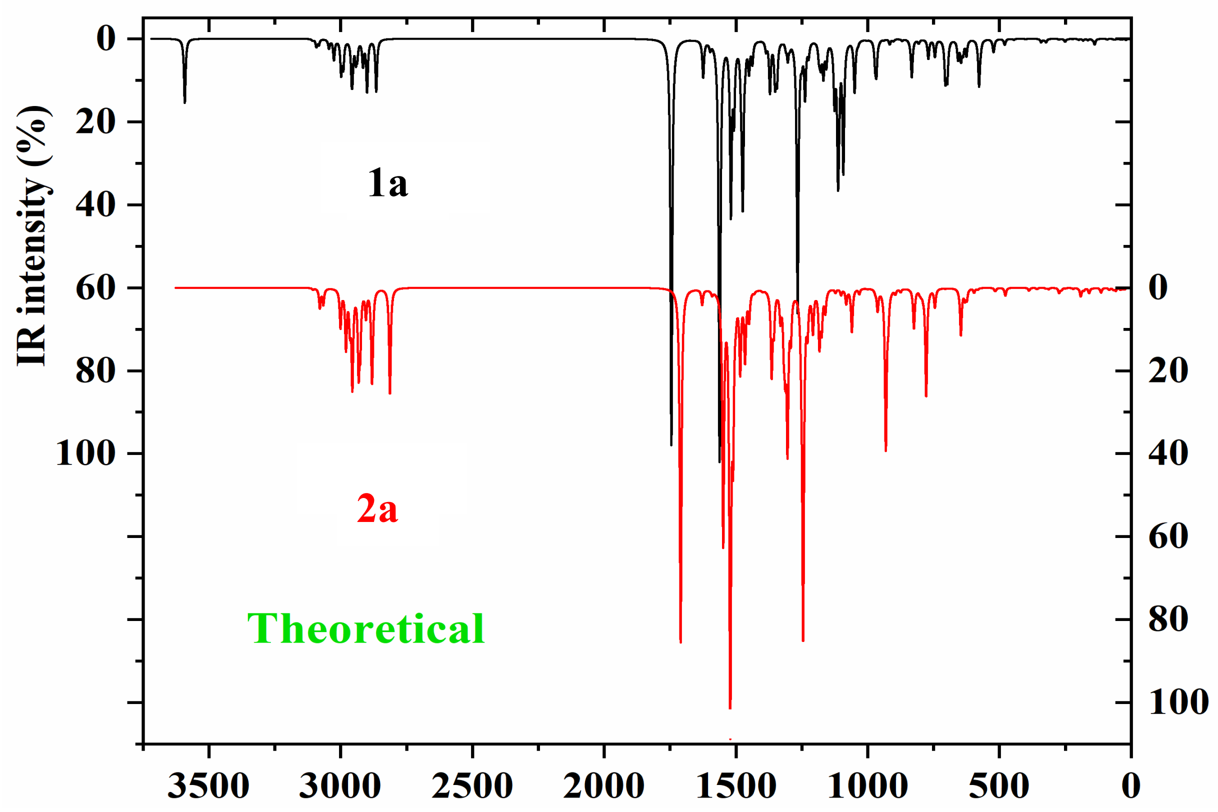

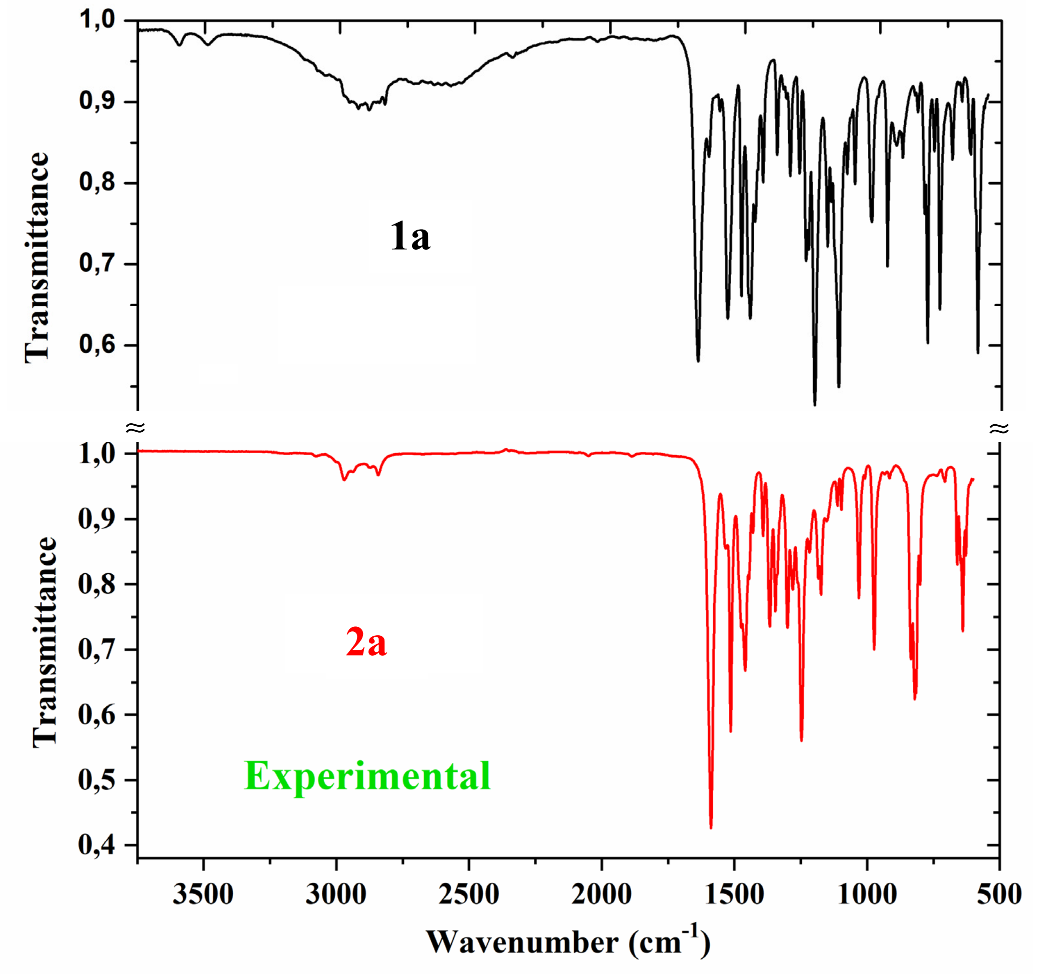

Figure S2-SUP. Comparison of the theoretical scaled IR spectra in the 3750-0 cm^{-1} range with the experimental ones in the 3750-500 cm^{-1} range of **1a** and **2a** molecules.

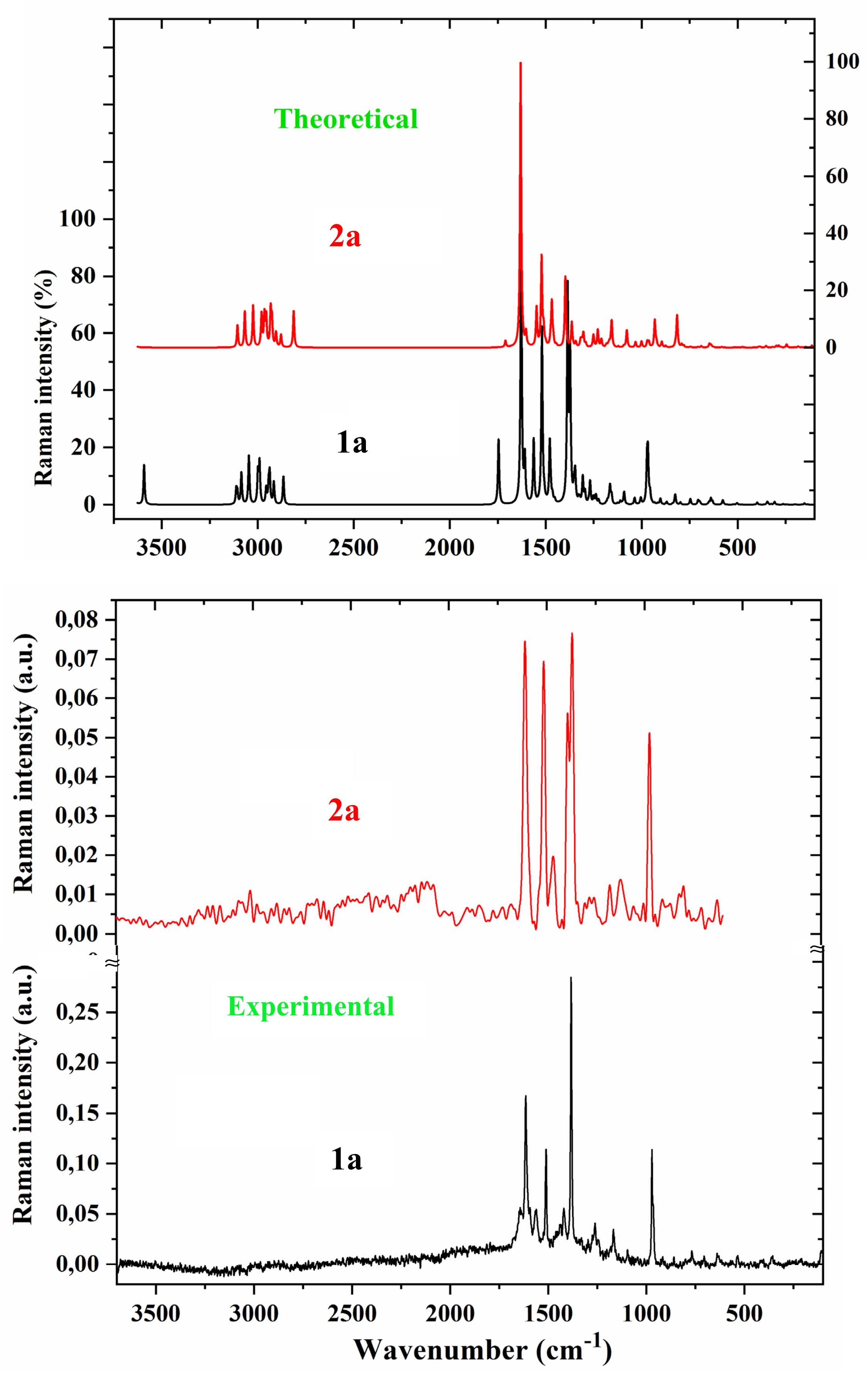

Figure S3-SUP. Comparison of the theoretical scaled IR spectra with the experimental ones in the 3750-100 cm^{-1} range of **1a** and **2a** molecules.

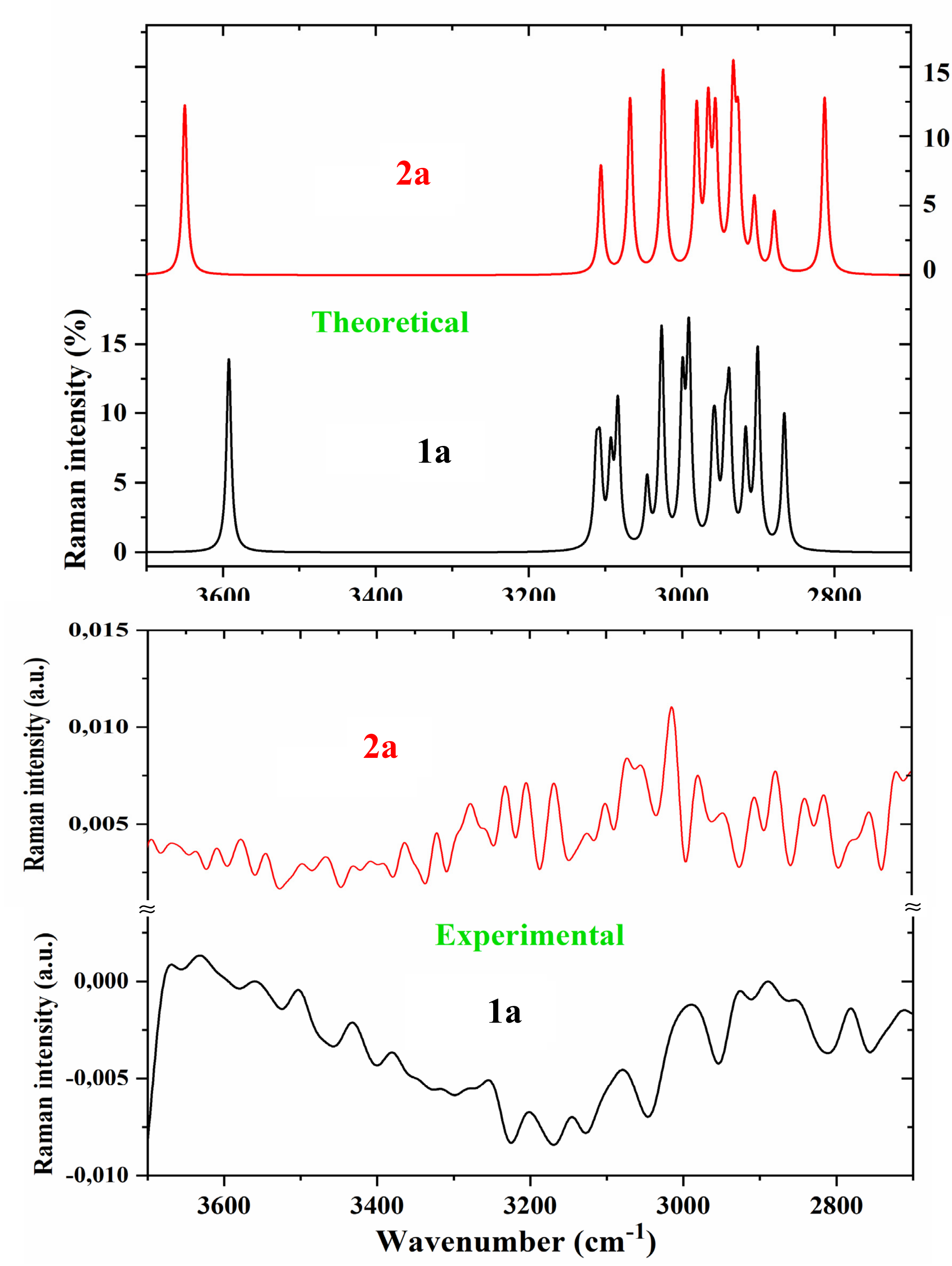

Figure S4-SUP. Comparison of the theoretical scaled Raman spectra with the experimental ones of **1a** and **2a** molecules in the 3700-2700 cm^{-1} range.

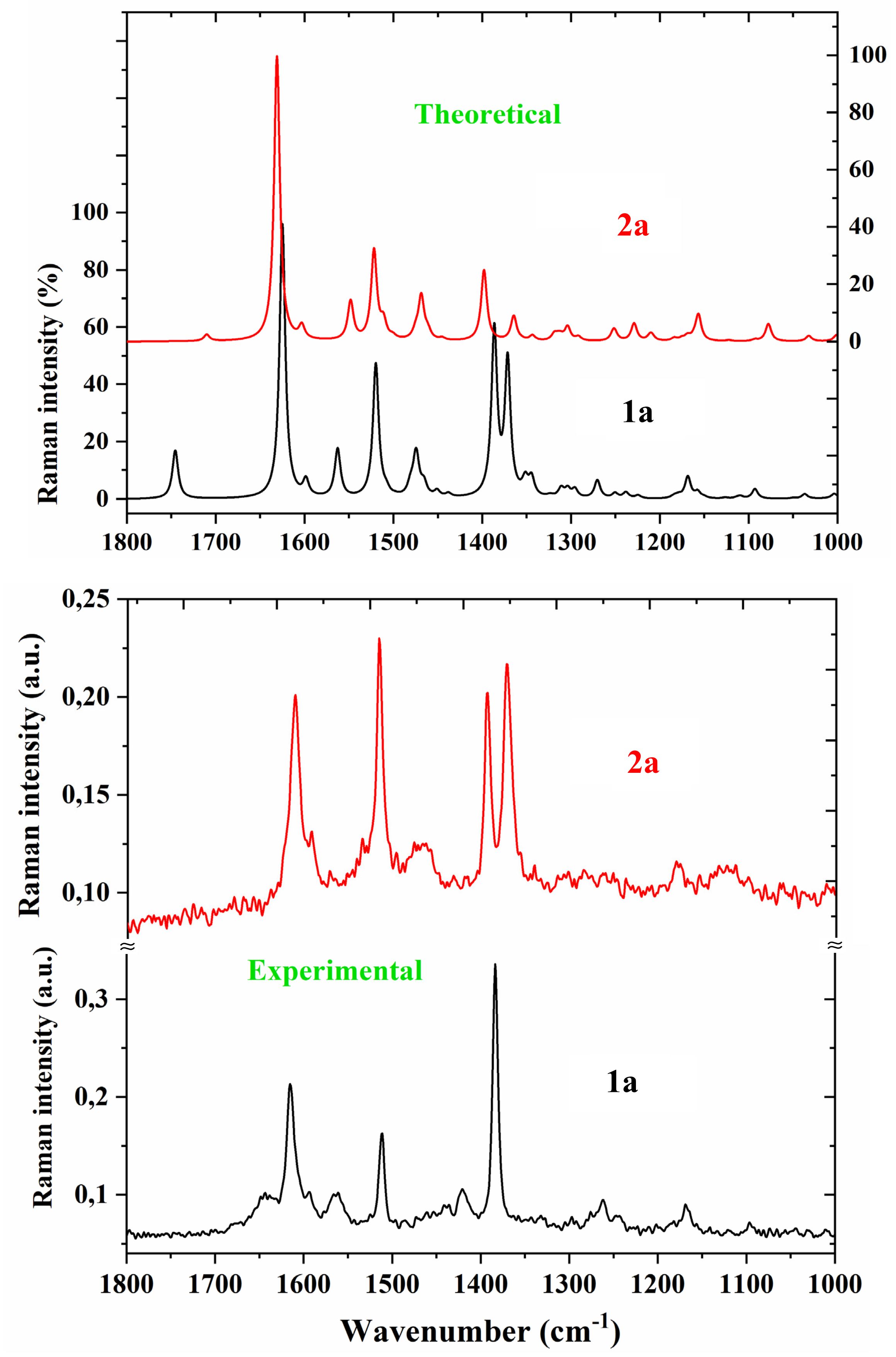

Figure S5-SUP. Comparison of the theoretical scaled Raman spectra with the experimental ones of **1a** and **2a** molecules in the 1800-1000 cm^{-1} range.

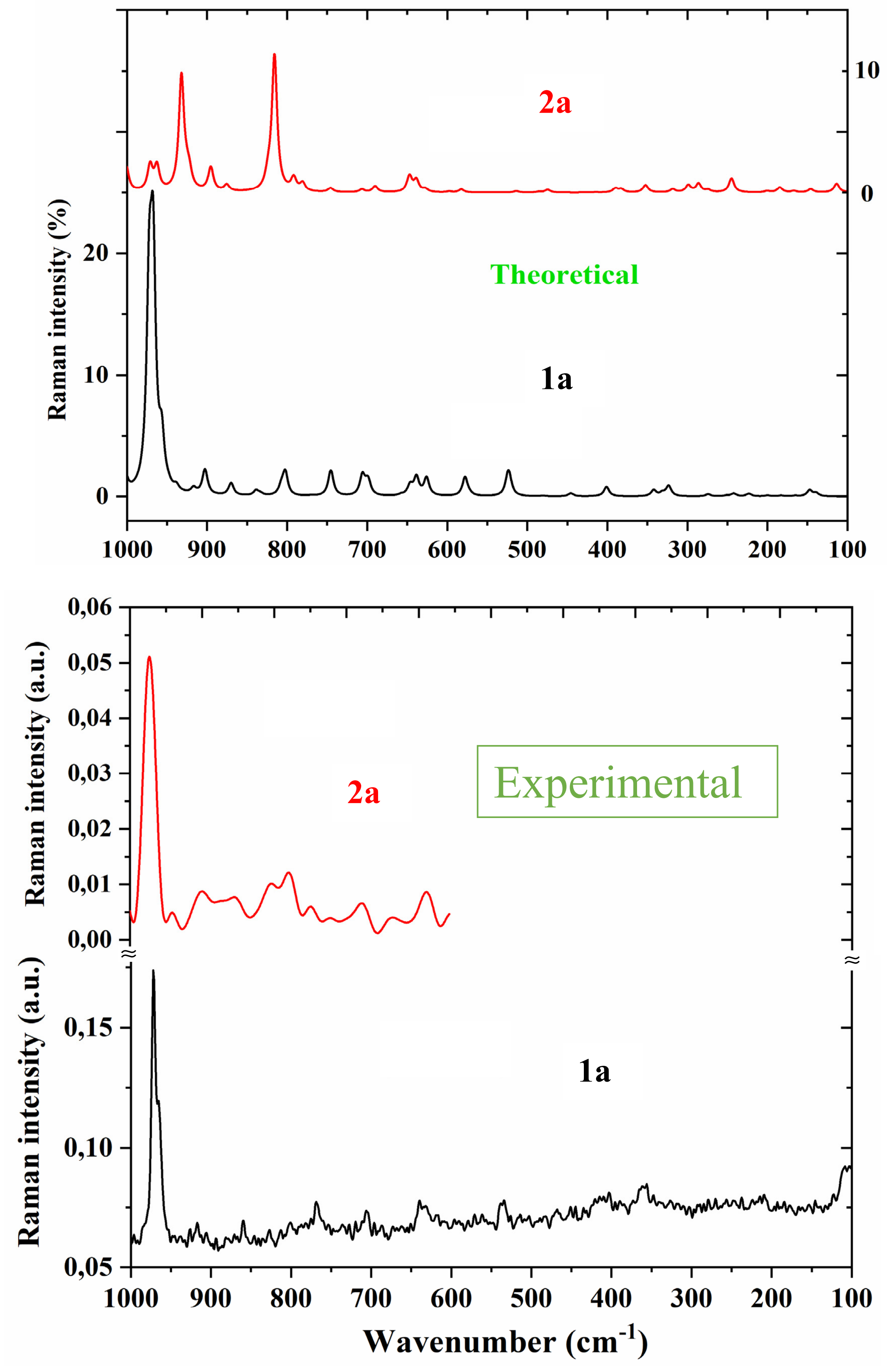

Figure S6-SUP. Comparison of the theoretical scaled Raman spectra with the experimental ones of **1a** and **2a** molecules in the 1000 – 0 and 1000-100 cm^{-1} ranges, respectively.

Table S1-SUP. Several selected optimized geometrical parameters calculated in the monomer form with the 6-31G(d,p) basis set. Bond lengths (r) in Å, bond angles and dihedral angles (∠) in degrees.

	-OCH_3				CL	-OH	-ONH_2	-OCF_3
	B3LYP		MP2		MP2	MP2	MP2	MP2
Parameters	**1a**	**2a**	**1a**	**2a**	**1b**	**1c**	**1d**	**1e**
r(C_4-N_4)	1.421	1.398	1.418	1.401	1.417	1.418	1.419	1.417
r(N_4-N_7)	1.349	1.357	1.340	1.348	1.340	1.340	1.340	1.340
r(N_4-N_{10})	1.311	1.343	1.334	1.350	1.334	1.334	1.334	1.334
r(C_8-C_9)	1.440	1.448	1.422	1.427	1.423	1.422	1.422	1.424
r(C_9-N_{10})	1.347	1.329	1.358	1.350	1.357	1.358	1.358	1.356
r(C_9-C_{11})	1.465	1.549	1.466	1.541	1.467	1.466	1.466	1.468
r(CL/O_1···O_{12})	9.934	9.857	9.895	9.872	10.227	9.889	9.898	9.875
O_{12}…H_{18}	2.152	1.980	2.181	2.030	2.186	2.181	2.180	2.188
∠(C_4-N_4-N_7)	121.6	122.4	121.5	121.9	121.5	121.5	121.5	121.5
∠(C_4-N_4-N_{10})	122.5	122.7	121.7	122.0	121.7	121.7	121.8	121.7
∠N-N-N)	115.9	115.0	116.7	116.1	116.8	116.7	116.7	116.8
∠(N_{10}-C_9-C_{11})	119.7	121.0	119.2	120.7	119.2	119.2	119.3	119.2
∠(N_7-C_8-N_{14})	119.4	119.5	119.6	119.1	119.6	119.6	119.6	119.6
∠(C_9-C_8-N_{14})	133.2	131.0	132.6	131.0	132.7	132.6	132.6	132.7
∠(C_9-C_{11}=O_{12})	126.1	113.9	125.7	113.6	125.6	125.7	125.7	125.6
∠(C_9-C_{11}-O_{13})	112.4	115.7	111.9	115.3	111.8	111.9	111.9	111.8
∠(O=C=O)	121.4	130.3	122.4	131.0	122.6	122.4	122.4	122.6
∠(C_5-C_4-N_4-N_{10})	-1.6	-1.1	-14.2	-3.0	-10.3	-13.7	-14.9	-10.7
∠(C_4-N_4-N_{10}-C_9)	-179.0	-178.5	-179.2	-178.8	-178.9	-179.1	-179.2	-179.0
∠(N_4-N_{10}-C_9-C_{11})	175.6	175.3	173.6	176.1	173.7	173.6	173.6	173.7
∠(N_{10}-N_4-N_7-C_8)	-0.4	-1.1	-0.9	-1.4	-0.8	-0.9	-0.9	-0.8
∠(N_{10}-C_9-C=O_{12})	-165.9	-145.7	-157.1	-149.3	-156.3	-157.1	-157.3	-156.1
∠(N_{10}-C_9-C-O_{13})	13.0	32.8	21.9	29.8	22.6	21.9	21.7	22.9
∠(C_8-C_9-C=O_{12})	9.6	29.1	15.0	26.5	16.0	15.0	14.7	16.4
∠(C_{11}-C_9-C_8-N_{14})	4.8	4.4	8.0	2.5	8.0	8.0	8.1	8.0
∠(C_9-C_8-N_{14}-C_{15})	178.2	177.8	173.2	172.5	172.9	173.2	173.3	172.7
∠(C_9-C_8-N_{14}-C_{18})	19.7	22.2	26.4	35.6	24.9	26.4	27.0	24.4
∠(C_8-N_{14}-C_{15}-C_{16})	-163.0	-169.6	-151.8	-163.7	-152.0	-151.8	-151.7	-152.0
∠(N_{14}-C_{15}-C_{16}-C_{17})	-21.5	-14.0	-22.9	-5.1	-23.6	-22.9	-22.6	-23.8

Table S2-SUP. Calculated harmonic wavenumbers (ν, cm^{-1}), scaled (ν, cm^{-1}) wavenumbers obtained and the linear scaling equation procedure (LSE) and the polynomic scaling equation procedure (PSE), experimental (Exp) wavenumbers, relative infrared intensity (A) in % and relative Raman intensity (S) in % obtained at the B3LYP/6-31G(d,p) level in **1a** and **2a** molecules. In the main characterization of the different bands, the number of the ring mode corresponds to Wilson's notation [17].

	1a								2a							
No	ν^{cal}	TLSE ν^{scal}	PSE ν^{scal}	A	S	Exp IR	Exp Raman	Characterization	ν^{cal}	TLSE ν^{scal}	PSE ν^{scal}	A	S	EXP IR	EXP Raman	Characterization
1	3762	3607	3592	15	17	3596.1 w		ν(O$_{13}$-H) in COOH (100)		--	--	--	--			
						3491.0 w	3504.2 vw	ν(O$_{13}$-H) H-bonded in COOH		--	--	--	--			
2	3243	3113	3112	0	6	3127.4 vw	3110.8 vw	2, ν(C$_3$-H) in aryl (100)	3237	3108	3106	0	7			2, ν(C$_3$-H) in aryl (100)
3	3238	3109	3107	0	6	3102.3 vw	3107.0 vw	20b, ν(C$_5$-H) in aryl (100)	3236	3107	3105	0	3		3125.3 vw	20b, ν(C$_5$-H) in aryl (100)
4	3223	3094	3093	2	6			20a, ν(C$_6$-H) in aryl (100)	3208	3080	3079	5	9	3078.2 vw		20a, ν(C$_6$-H) in aryl
5	3213	3085	3084	1	10	3083.0 vw	3076.1 vw	7b, ν(C$_2$-H) in aryl (100)	3194	3067	3066	4	12		3075.1 vw	7b, ν(C$_2$-H) (100)
6	3172	3046	3045	2	5	3053.1 vw	3067.4 vw	ν_{as}(C-H) in C18H$_2$ pyrrolidine (100)	3124	3000	3001	9	17		3014.4 vw	ν_{as}(C-H) in CH$_3$ (100)
7	3151	3026	3026	5	16	3044.5 vw	3025.9 vw	ν_{as}(C-H) in CH$_3$ (100)	3102	2979	2980	13	12		2982.6 vw	ν_{as}(C-H) in phase in C16H$_2$,C17H$_2$ (100)
8	3122	2998	2999	8	11	3009.7 vw	3018.0 vw	ν_{as}(C-H) in C16H$_2$, C17H$_2$ pyrroli (100)	3086	2964	2965	8	12		2975.8 vw	ν_{as}(C-H) in C16H$_2$, C17H$_2$ pyrrolidin(100)
9	3113	2990	2990	6	15	2985.6 w	2995.1 vw	ν_{as}(C-H) in C16H$_2$, C17H$_2$ pyrroli(100)	3076	2955	2956	22	11	2969.2 w	2951.7 vw	ν_{as}(C-H) in C18H$_2$ pyrrolidine (100)
									3051	2931	2933	8	13	2938.4 sh	2941.1 vw	ν_s(C-H) in C17H$_2$ pyrrolidine (100)
10	3079	2958	2959	7	6	2964.4 w	2968.1 vw	ν_{as}(C-H) in CH$_3$ (100)	3050	2930	2932	10	6			ν_{as}(C-H) in CH$_3$ (100)
11	3076	2955	2956	6	5	2953.8 vw		ν(C-H) in C15H$_2$, C16H$_2$ pyrrolidi (100)	3044	2924	2926	12	9		2911.2 vw	ν_s(C-H) in C16H$_2$ pyrrolidine (100)
12	3062	2941	2943	4	6		2941.1 vw	ν(C-H) in pyrrolidine (100)								
13	3056	2936	2937	4	10	2931.6 w	2928.6 vw	ν(C-H) in pyrrolidine (100)	3021	2902	2905	6	5			ν_{as}(C-H) in C15H$_2$ pyrrolidine (100)
14	3033	2914	2916	6	8	2917.2 w	2922.8 vw	ν_s(C-H) in C15H$_2$ pyrrolidine (100)	2997	2880	2882	17	20	2870.9 vw	2898.7 vw	ν_s(C-H) in CH$_3$ (100)
15	3017	2899	2901	12	14	2892.1 w	2897.7 vw	ν_s(C-H) in CH$_3$ (100)	2994	2877	2879	7	4	2841.9 w	2880.3 vw	ν_s(C-H) in C15H$_2$ pyrrolidine (100)
16	2979	2862	2865	12	10	2866.1 w		ν_s(C-H) in C18H$_2$ pyrrolidine (100)	2924	2810	2814	24	13	2673.9 vw	2808.0 vw	ν_s(C-H) in C18H$_2$ pyrrolidine (100)
	--	--	--	--	--	2834.2 w		ν(O-H)$_{H\text{-}bonded}$, Combination band	--	--	--	--	--	2359.7vw	2638.3 vw	Combination bands
		--	--	--	--	2726.2, 2623.0	2723.2 vw	ν(O-H)$_{H\text{-}bonded}$, Combination band		--	--	--	--	2284.6vw		Combination bands
17	1796	1738	1745	97	18	1675.1 vs	1643.2 m	ν(C=O) (92)	1759	1702	1710	83	2	1588.3 vs	1629.7 vw	ν_{as}(COO) (96)
18	1670	1618	1625	9	100	1634.6 w	1615.2 vs	8a, ν(C=C) (96)	1674	1622	1629	3	100		1610.4 vs	8a, ν(C=C) (95)
19	1643	1592	1599	2	6	1594.0 vw	1594.0 m	8b, ν(C=C) (97)	1634	1583	1590	1	2		1592.1 m	8b, ν(C=C) (89)
20	1605	1556	1562	100	17	1565.1 vs	1561.2 m	ν(C$_8$-N$_{14}$) (65) + ν_s(N$_7$CC) (20)	1591	1543	1549	58	12	1533.3 w	1535.2 w	ν(C$_8$-N$_{14}$) (72) + ν_s(N7CC) (15)
21	1561	1514	1520	40	48	1514.0 vs	1511.1 s	19a, ν(CC)(87)+δ_s(CH) pyrrolidi (11)	1563	1516	1522	100	23	1514.0 vs	1515.9 vs	19a, ν(CC) (76)+δ_s(CH)pyrrolidine(18)
22	1548	1502	1507	17	2		1524.6 w	δ_s(C-H) C15H$_2$, C18H$_2$ in-phase(98)	1552	1505	1511	32	5		1504.3 sh	δ_s(C-H) in C15H$_2$, C18H$_2$ in phase (84)
23	1537	1491	1497	0	0			δ_s(C-H)C15H$_2$, C18H$_2$ out-of-phase (100)	1541	1495	1501	2	1		1496.6 w	δ_s(C-H) in C15H$_2$, C18H$_2$ out-of phase(97)
24	1520	1475	1481	7	3	1488.2 sh	1496.6 vw	δ_s(C-H) in CH$_3$ (100)	1524	1479	1484	18	1			δ_s(C-H) in CH$_3$ (97)
25	1515	1470	1476	22	6	1482.2 vs	1485.1 w	δ_s(C-H) out-of-phase pyrrolidine (83)	1514	1469	1475	1	2	1474.5 m	1476.4 m	δ_s(C-H) out-of-phase in pyrrolidine (92)
26	1513	1468	1474	17	10	1463.9 s	1472.5 w	δ_s(C-H) out-of-phase pyrrolidine (75)	1505	1461	1466	15	13	1460.0 s	1466.7 m	ν_s(C$_8$C$_9$N)(62)+δ_s(CH$_2$) (16)+ν(NN)(15)
27	1505	1461	1466	1	3		1461.0 w	δ_{as}(C-H) in CH$_3$ (100)	1500	1456	1461	0	2			δ_s(C-H) out-of-phase in pyrrolidine (97)
28	1504	1460	1465	1	2	--	--	δ_s(C-H) out-of-phase pyrrolidine (98)	1500	1456	1461	1	3	--	1462.9 m	δ_{as}(C-H) in CH$_3$ (99)
29	1489	1446	1451	7	2			δ_s(C-H) in CH$_3$ (87)	1488	1445	1450	7	2	1444.6 sh	1448.4 w	δ_s(C-H) in CH$_3$ (87)
30	1476	1433	1438	5	1	1434.0 m	1420.5 m	19b, ν(CC,CH) in aryl (72)	1469	1427	1431	1	0	1430.1 w		19b,ν(CH,CC) in aryl (65) + ν_s(C8C9N)(14)

31	1422	1382	1386	2	57		1383.8 s	ν_s(NNN)(35)+ν_s(C4N) (28) +ν(COO)(25)	1435	1394	1399	0	20	1392.5 w	1393.4 vs	ν(C4N)(41) +ν(NNN)(25) +19a,ν(CC)(15)
32	1407	1368	1372	12	46	1381.9 m	1362.6 vw	ν(C4N) (28) +ν(NNN)(22) +δ(COO) (18)	--	--	--	--	--	--	--	--
33	1389	1351	1354	2	0	1357.8 vw	1357.8 vw	ν(C9N)(32) +δ(COO)(25) +δ_s(pyrrol) (23)	1400	1361	1365	19	7	1367.4 m	1372.2 vs	γ_s(C-H) in-phase in pyrrolidine (87)
34	1386	1348	1351	8	5	1348.2 vw	1349.1 vw	γ_s(C-H) in pyrrol (65)+ν(C-OH) (15)	1391	1352	1356	8	0	1346.2 m	1356.8 w	ν_s(NNN)(38)+γ_s(C-H)in pyrrol(30)+ν(C8C)(25)
35	1379	1341	1345	9	6	1333.7 m	1343.3 vw	14,ν(CC) aryl (60) + ν_s(NNN) (25)	1365	1328	1331	6	1	1339.5 m	1340.4 vw	14,δ(CC) in aryl (82) + δ(CH$_3$) (16)
36	1357	1320	1323	0	1		1331.7 w	γ_s(C-H) in pyrrolidine (83)	1352	1315	1319	10	1		1326.0 vw	ν(CN,CC)triazole(34) +ν_s(COO)(28)+γ(CH)pyrrol(25)
37	1344	1308	1311	1	3		1327.9 w	γ_{as}(CH) pyrrolidi (48)+3,δ(CH) in aryl(38)	1346	1310	1313	14	1		1314.4 vw	γ_{as}(C-H) pyrrolidine (46) + ν_s(COO) (37)
	--	--	--	--	--	--	--	--	1337	1301	1304	35	4	1299.9 m	1307.6 vw	ν_s(COO)(34)+ν(triazole)(32)+γ(CH) pyrrolid(18)
38	1337	1301	1304	4	3	1299.0 m	1304.7 vw	3,δ(CH) (50) +γ_s(C-H) pyrrolidine (32)	1332	1296	1299	1	0		1302.8 vw	3,δ(CH) in aryl (65)+γ(CH) pyrrolidine (16)
39	1328	1292	1295	1	3		1297.0 vw	γ_s(C-H) in pyrrolidine (83)	1324	1289	1291	9	1	1280.7 m	1285.5 vw	γ_s(C-H) in-phase in pyrrolidine (93)
40	1302	1268	1270	1	6		1275.8 w	ν(CN) triazol (56) +3,δ(CH) aryl(23)	--	--	--	--	--	--	--	--
41	1298	1264	1266	64	0	1274.9 s	1262.0 m	ν(C-O1) (65) +14,ν(CC) in aryl (22)	--	--	--	--	--	--	--	--
42	1282	1249	1251	3	2	1265.2 s	1246.9 w	γ_{as}(C-H) in pyrrolidine (78)	1283	1250	1252	9	2	1262.3 sh	1260.4 vw	γ_{as}(C-H) in pyrrolidine (92)
43	1269	1236	1238	13	2	1243.1 vs	1243.0 w	δ(OH) (52)+ν(CN)(25)+γ_{as}(C-H)(16)	1276	1243	1245	79	1	1247.9 vs	1251.7 vw	7a,ν(COC)(62)+δ(CH)aryl(25)+γ_{as}(CH$_3$)(11)
44	1255	1223	1225	3	1		1222.8 vw	γ_{as}(C-H) in pyrrolidine (85)	1259	1227	1229	8	5	1217.0 vw	1237.2 vw	δ(NN)(37)+ν(CC11)(25)+δ(CC,CN)pyrrol(24)
	--	--	--	--	--	--	--	--	1241	1210	1211	0	1		1213.1vvw	γ_{as}(C-H) in pyrrolidine (92)
45	1215	1185	1186	3	0	1194.8 s	1201.6 vw	γ_{as}(C-H) in pyrrolidine (88)	1238	1207	1208	9	1		1208.3vvw	δ(triazole)(45)+γ(CC,CH)pyrrolidi(25)+3,δ(CH)(13)
	--	--	--	--	--	--	--	--	1213	1183	1184	8	0	1184.2 m		δ(triazole)(73)+γ(CC,CN)pyrrolidine(16)
46	1212	1182	1183	2	1	1180.4 m	1182.3 w	γ_{as}(C-H) in CH$_3$ (89)	1211	1181	1182	5	1		1180.4 m	γ_{as}(C-H) in CH$_3$ (92)
47	1207	1177	1178	5	1	1153.4 vs	1175.5 sh	γ_{as}(C-H) pyrrolidin (82)+δ(O13H)(14)	1203	1174	1174	8	1	1173.6 m	1175.5 m	γ_{as}(C-H) pyrrolidine(68) +δ(triazole) (15)
48	1197	1168	1169	8	7	1166.9 sh	1168.8 m	9a, δ(C-H) in aryl (84)	1191	1162	1163	0	9		1167.8 w	9a, δ(C-H) in aryl (78)
49	1185	1157	1157	5	2		1156.2 w	γ_{as}(CH)pyrrolidin(65)+9a,δ(C-H) (23)	1189	1160	1161	5	1		1164.0 vw	γ_{as}(C-H) pyrrolidine (62)+9a,δ(C-H) (28)
50	1178	1150	1150	0	0			γ_{as}(C-H) in CH$_3$ (99)	1180	1152	1152	0	0	1152.4 w	1154.3 w	γ_{as}(C-H) in CH$_3$ (92)
51	1154	1127	1127	14	0		1140.8 w	ν_s(COOH)(45)+δ_{as}(CH) pyrrolidi (42)	1149	1122	1122	1	0	1111.9 vw	1123.5 m	δ_{as}(C-H) in pyrrolidine (93)
52	1140	1114	1113	29	0	1122.5 m	1120.6 w	ν_{as}(COO)(33)+ν_s(NNN)(31) +15,δ(CH)(28)	--	--	--	--	--	--	--	--
53	1136	1110	1109	10	1	1108.0 vw	1106.1vvw	15, δ(C-H) in aryl (68)+ν_s(COO)(15)	1128	1102	1102	1	0	1096.5 vw	1099.4 w	15, δ(C-H) in aryl (96)
54	1119	1094	1093	30	3	1093.6 m	1096.5 w	ν_s(COO) (45) + δ(NNN) (38)	1107	1082	1081	3	3			18b,δ(CH) in aryl (43) +ν(triazole)(40)
55	1075	1052	1050	12	0			ν(O1-CH$_3$) (83)	1085	1061	1060	10	4		1063.7 w	ν_{as}(COC)(62)+15,δ(CH)(17)+γ_s(CH$_3$)(15)
56	1061	1039	1037	1	1	1031.9 m	1044.4 vw	γ_s(CC, CH) in pyrrolidine (82)	1056	1034	1032	1	1	1030.9 m	1047.3 w	γ_s(CC,C-H) in pyrrolidine (82)
57	1027	1006	1004	0	1	1006.7 vw	1010.6 vw	18a, δ(CH, CC) in aryl (88)	1024	1003	1001	0	1	1008.7 vw	1010.6 w	18a, δ(CC, CH) in aryl (96)
58	995	980	973	5	9	973.0 s	972.1 s	ν_{as}(NNN)(42)+δ(CN14)(24)+γ(CC) pyrrol(22)	994	975	972	1	2	973.0 m	975.0 vs	ν_{as}(NNN)(32)+δ(CN14)(29)+δ(CC) pyrrol(25)
59	990	975	968	7	11		965.3 m	ν_s(NNN)(38)+12,δ(CC)(34)+δ(CC,CN)(24)	985	966	963	5	1		962.4 w	γ(C-H) in pyrrolidine (96)
60	979	964	957	0	2			δ(CC,CN) in pyrrol(58)+ν_s(NNN)(36)	--	--	--	--	--	--	--	--
61	971	956	949	0	0		948.0vvw	17a, γ(C-H) in aryl (99)	959	942	938	0	0		945.1 vw	17a, γ(C-H) in aryl (100)
62	960	945	939	0	0	939.3 m	937.4 vw	5, γ(C-H) in aryl (100)	953	936	932	5	1	933.4 vvw	927.7 vw	5, γ(C-H) in aryl (100)
63	938	923	917	1	0	925.8 vw	925.8 vw	γ_{as}(C-H) in pyrrolidine (98)	953	936	932	32	6			ν_s(NNN)(44)+ν(C8C)(20)+10b,γ (CH)(18)
64	923	908	902	1	1	917.1 m	917.1 w	γ_{as}(CC, CH) in pyrrolidine (97)	943	926	922	6	1	916.1 vvw	915.2 vw	γ_{as}(C-H) in pyrrolidine (99)
65	890	875	870	0	1	860.2 w	860.2 w	γ_s(CC, CH) in pyrrolidine (93)	916	901	896	1	1		907.5 w	γ_s(C-H) in pyrrolidine (100)
66	858	843	839	0	0		845.8 vw	γ_{as}(CC, CH) in pyrrolidine (87)	895	881	875	1	0		867.0 w	γ_{as}(C-H) in pyrrolidine (100)
67	852	837	833	9	0	835.1 m	827.4 w	17b, γ(C-H) in aryl (98)	843	831	825	9	0	835.1 m	821.7 w	17b, γ(C-H) in aryl (100)
	--	--	--	--	--	--	--	--	843	831	825	1	0	820.7 m	816.8 w	δ_{as}(C-H) in pyrrolidine (99)
68	825	810	807	1	0	824.5vs	813.0 w	10a, γ(C-H) in aryl (97)	821	810	803	1	0	814,9 m	801.4 m	10a, γ(C-H) in aryl (100)
69	820	805	802	0	1	799.4 m	800.4 w	6a,δ(CC)aryl (51) +δ_s(CO1C) (25)	815	805	797	1	4	800.4 w	797.5 m	6a,δ(CC)(56)+γ(CH$_3$)(25)+ δ(CH) pyrroli (18)
70	787	772	770	5	0	779.2 vs	768.0 m	δ(CCOO) (67) + γ(C8C) (22)	808	798	790	1	1		783.1 vw	γ_s(CCOO)(55)+γ(C8C)(30) +6a,δ(CC)(27)

	--	--	--	--	--	--	--	--	795	786	778	25	1		774.4 w	δ_{as}(COO) (58)+ δ_{as}(C-H) pyrrolidine (15)
71	762	747	745	4	1	732.9 m	735.8 vw	δ(CC,CH) in pyrrol(70) +δ(CCOO) (17)	762	754	745	4	0	737.7 vvw	730.0 w	δ(CC) in pyrrolidine (65) + δ_{s}(COO) (25)
72	721	706	705	9	1	707.8 vvw	705.9 w	γ(COOH)(62)+γ(NC8C)(21)+γ(CN14)(16)	722	716	706	0	0	706.9 vw	707.9 w	γ(NC8CN10) (53) + γ_{s}(COO) (38)
73	716	701	701	0	0			10b, γ(CC, CH) in aryl (97)	710	705	695	0	0	660.6 w	671.2 vw	10b, γ(CC, CH) in aryl (99)
74	714	699	699	9	0	697.2 w	696.3w,686.7w	δ(COOH) (46) + γ(triazole) (38)	--	--	--	--	--	--	--	--
75	672	657	657	4	0	664.4 m	662.5 vw	γ_{s}(triazole) (78) + γ(O13-H) (15)	--	--	--	--	--	--	--	--
76	661	646	647	4	0	647.1 sh	651.0 w	6a, δ(CC) (38) + Γ(triazole) (32)	661	658	647	11	1	648.0 w	642.3 vw	6a,δ(CC)(65)+δ(NNN)(15)+δ(OCH_3)(12)
77	652	637	638	3	1	638.4 vs	639.2 m	6b, δ(CC) (42)+ δ(triazole) (24)	646	644	632	2	1	639.4 m	637.5 w	6b, δ(CC) in aryl (68) +δ(NNN)(15)
78	640	625	626	4	1	614.3 vvw	631.7 m	Γ(triazole)(34)+6b,δ(CC) in aryl(33)	638	636	624	2	0	627.8 w	627.8 w	Γ(triazole) (38) + δ(CC) in aryl (25)
79	591	576	578	11	1		570.9 w	γ(O13-H) (81)	610	610	597	1	0	613.3 vvw	607.6 w	γ_{s}(NNN) (55) + γ(COO) (18)
80	584	569	571	1	0		561.3 w	δ(CC, CH) in pyrrolidine (94)	595	595	582	0	0			δ_{as}(CC, C-H) in pyrrolidine (100)
		--	--	--	--		--	--	--	--	--	--	--			--
81	536	520	524	1	1		534 6 m	16b,γ(CC)(45)+δ(COC)(18)+δ(triazole)(24)	529	533	517	1	0			16b. γ(CC,CH) in aryl (86)
82	534	518	522	2	0		502.5 w	6b,δ(CC) (38)+δ(COC)(26) +δ(COO)(22)	525	529	513	0	0			6b,δ(CC)(38)+δ(triazole)(32) +δ(OCH_3)(18)
83	491	475	480	1	0		467.8 vw	δ(triazole) (34)+ 6b,δ(CC) (30)+δ(COO)(24)	488	494	477	2	0			δ(triazole)(42) +6b,δ(CC)(22)+δ(COO)(17)
84	456	440	446	0	0			16a, γ(CC) (28)+δ(COC)(26)+δ(triazole)(24)	442	450	432	0	0			16a,γ(CC)(46)+δ(triazole)(30) +δ(COO) (12)
85	425	409	415	0	0		409.9 w	4, γ(CC) in aryl (85)	427	436	417	0	0			4, γ(CC) in aryl (95)
86	411	395	401	0	0		403.2 w	Γ(CC) aryl(32) +δ(triazole)(28) + δ(COC)(26)	397	407	388	1	0			
		--	--	--	--		389.7 vw			--	--	--	--			
	351	335	342	1	0		361.7 m		367	379	358	0	0			
	340	324	332	0	0		355.9 m		331	344	323	0	0			
	332	316	324	1	0		327.0 vw		321	335	313	0	0			
	282	266	274	0	0		310.6 vw		281	297	274	1	0			
	259	243	252	1	0		270.1 vw		272	288	265	1	0			
	249	233	242	0	0		237.3 vw		256	273	249	0	0			
	230	214	223	0	0		215.1 vw		227	246	220	0	1			
	206	190	200	0	0		209.3 vw		198	218	192	2	0			
	189	173	183	0	0		184.3 vw		186	207	180	1	0			
	171	155	165	0	0		145.7 vw		165	187	159	1	0			
	153	137	147	0	0		122.6 vw		151	173	145	0	0			
	145	129	139	1	0				120	144	115	1	1			
	97	81	92	0	0				89	114	84	1	0			
	71	55	66	0	0				72	98	67	0	0			
	66	50	61	0	0				64	91	59	1	0			
	46	30	41	0	0				61	88	56	0	0			
	44	28	39	0	0				45	72	40	0	0			
	37	21	32	0	0				36	64	31	0	0			
	25	9	21	0	0				29	57	25	0	0			

*Assigned to the dimer form. †Observed frequencies characterized by notation: vs= very strong, s= strong, m= medium, ms= medium strong, w=weak band, vw= very weak, vvw= very very weak, sh= shoulder. ν_{as}: anti-symmetric stretching, ν_{s}: symmetric stretching, δ: in-plane bending, γ: out-of-plane bending, β: scissoring, ρ: rocking, ω: wagging, τ: torsion

Table S3-SUP. Calculated harmonic wavenumbers (ν, cm^{-1}), relative infrared intensities (A, %), relative Raman intensities (S, %) together with the scaled values (ν, cm^{-1}) in the COOH (**1a**) and COO groups (2a).

Group					TLSE	PSE			
		ν^{cal}	A	S	ν^{scal}	ν^{scal}	IR	Raman	Characterization
COOH	ν(O-H)	3762	15	17	3607	3592	3596.1 w		ν(O-H) (100)
		3161	100	0	3035	3035	3491.0 w	3504.2 vw	Dimer: ν(O-H) out-of-phase
		3062	0	100	2941	2943			Dimer: ν(O-H) in-phase
	δ(O-H)	1269	13	2	1236	1238	1243.1 vs	1243.0 w	δ(O-H)(52) + ν(CN)(25) + γ_{as}(CH) in pyrrolidine (16)
	γ(O-H)	591	11	1	576	578		570.9 w	γ(O-H) (81)
		1015	3	0	1001	998			Dimer: γ(O-H) in-phase
		964	0	0	949	942			Dimer: γ(O-H) out-of-phase
	ν(C=O)	1796	97	18	1738	1745			ν(C=O) (92)
		1743	80	0	1687	1695	1675.1 vs		Dimer: ν(C=O) out-of-phase
		1702	0	70	1648	1655		1643.2 m	Dimer: ν(C=O) in-phase
	ν(C-O)	1140	29	0	1114	1113	1122.5 m	1120.6 w	ν_{as}(COO)(33) + ν_{s}(NNN)(31) + 15, δ(CH)(28)
		1286	0	1	1253	1255			Dimer: ν_{as}(COO) in-phase
		1285	2	0	1252	1254			Dimer: ν_{as}(COO) out-of-phase
		1119	30	3	1094	1093	1093.6 m	1096.5 w	ν_{s}(COO) (45) + δ(NNN) (38)
		821	1	0	806	803			Dimer: δ_{as}(COO) out-of-phase
		809	0	1	794	791			Dimer: δ_{as}(COO) in -phase
	δ(C=O)	714	9	0	699	699	697.2 w	696.3 w	δ(COOH) (46) + γ(triazole) (38)
		796	15	0	782	780	779.2 vs		Dimer: δ(COOH) out-of-phase + ν(CC)
		777	0	8	763	761		768.0 m	Dimer: δ(COOH) in-phase
	γ(C=O)	721	9	1	706	705	707.8 vvw	705.9 w	γ(COOH)(62) + γ(NC_8C)(21) + γ(CN_{14})(16)
		714	0	0	699	699			Dimer: γ(C=O) out-of-phase
		712	0	0	697	697			Dimer: γ(C=O) in-phase
COO	ν_{as}	1759	83	2	1702	1710	1588.3 vs	1629.7 vw	ν_{as}(COO) (96)
	ν_{s}	1337	35	4	1301	1304	1299.9 m	1307.6 vw	ν_{s}(COO)(34) +ν(triazole)(32) +γ(CH) in pyrrolidine (18)
	δ_{as}	795	25	1	786	778		774.4 w	δ_{as}(COO)(58) + δ_{as}(C-H) in pyrrolidine (15)
	γ_{s}	808	1	1	798	790		783.1 vw	γ_{s}(CCOO)(55) + γ(C_8C)(30) + 6a, δ(CC) (27)
